# A new laminopathy caused by an Arg133/Leu mutation in *lamin A/C* and the effects thereof on adipocyte differentiation and the transcriptome

**DOI:** 10.1080/21623945.2019.1640007

**Published:** 2019-07-11

**Authors:** Zhe Wang, Yueting Dong, Jing Yang, Yingzi He, Xihua Lin, Fang Wu, Hong Li, Fenping Zheng

**Affiliations:** a Department of Endocrinology, The Affiliated Sir Run Run Shaw Hospital, College of Medicine, Zhejiang University, Hangzhou, Zhejiang, China; b Biomedical Research Center and Key Laboratory of Biotherapy of Zhejiang Province, The Affliated Sir Run Run Shaw Hospital, Zhejiang University, Hangzhou, Zhejiang, China

**Keywords:** Laminopathy, lamin A/C, adipocyte differentiation, insulin resistance, DNA damage

## Abstract

We report a new laminopathy that includes generalized lipoatrophy, insulin-resistant diabetes, micrognathia and biopsy-proven, focal segmental glomerulosclerosis in a female, caused by a de novo heterozygous mutation R133L in the lamin A/C gene (*LMNA*). We analysed the nuclear morphology and laminar distribution in 3T3-L1 pre-adipocytes overexpressing human wild-type lamin A/C (*LMNA* WT) or lamin A/C with the R133L mutation (*LMNA* R133L). We found the nuclear size was varied, nuclear membrane invagination or blebbing, and an irregular A-type lamin meshwork in cells overexpressing *LMNA* R133L.3T3-L1 pre-adipocyte differentiation into adipocytes was impaired in cells expressing *LMNA* R133L; contemporaneously, the expression levels of genes associated with adipose tissue self-renewal, including adipogenesis, angiogenesis, and extracellular matrix maintenance, were downregulated. Furthermore, the insulin-signalling pathway was inhibited in *LMNA* R133L adipocytes. Microarray gene expression profiling showed that the most prominent differences between 3T3-L1 cells expressing wild-type *LMNA* and *LMNA* R133L were in genes implicated in metabolic pathways, the cellular response to DNA damage and repair. We thus expand the clinical spectrum of laminopathy and conclude that the *LMNA* R133L mutation associated with lipodystrophic features and multisystem disorders likely impairs adipocyte renewal and disrupts the expression of genes implicated in the induction and repair of DNA damage.

## Introduction

The *LMNA* gene is located on chromosome 1q21-22 and encodes the nuclear envelope proteins *lamin A* and *C* via alternative splicing; these, together with the type B lamins, are integral components of the nuclear laminae. Type B lamins are ubiquitously expressed, but expression of *lamin A* and *C* is (usually) developmentally regulated in both the lineage progenitors and differentiated cells. Lamins interact with the cytoskeleton, chromatin, transcription factors, and signalling molecules to become involved in nuclear assembly, the development of nuclear architecture and mechanical stability, and genome organization and stability. Mutations in *LMNA* trigger changes in nuclear architecture, signal transduction, and gene regulation, associated with a group of multisystem diseases termed laminopathies; these are characterized by lipodystrophy, muscular dystrophy, neuropathies, premature ageing syndromes, diabetes associated with insulin resistance, and (rarely) overlapping syndromes [1].

Obesity and lipodystrophy share similar metabolic defects associated with insulin resistance, illustrating the complex relationships between deregulation of adipose tissue and systemic metabolism. When nutrient intake exceeds the storage capacity of adipose tissue, lipids are ectopically deposited in non-adipose tissues, causing metabolic inflexibility. This is especially true of patients with lipodystrophy. A recent genomic study in a general population revealed that limited peripheral adipose storage capacity is a major determinant of insulin resistance [2]. Furthermore, the severity of metabolic complications seems to correlate with the extent of lipodystrophy [3].

The lamin functional domains are organized into a short N-terminal domain (the ‘head’); a central α-helical rod domain driving lamin dimerization and polymerization; and a large C-terminal tail containing the nuclear localization signal and an immunoglobulin-like folded domain with multiple binding sites. Mutations in the region encoding the C-terminal tail (exons 7–12) are usually associated with partial or generalized lipoatrophy, giving rise to Familial partial lipodystrophy (FPLD), A type mandibuloacral dysplasia (MAD-A), and Emery-Dreifuss muscular dystrophy (EDMA) [4–6]. It is well known that missense changes in the C-terminal half of *LMNA* trigger accumulation of pre-lamin A and change the expression of genes involved in adipocyte differentiation and proliferation (those encoding the peroxisome proliferator-activated receptor *PPARγ, cyclin D3*, retinoblastoma protein [*pRb*], and lipoprotein lipase) [7–9]. Although rare, mutations in the *LMNA* rod domain (exons 2–6) seem to be associated with a more severe phenotype in terms of generalized lipodystrophy and multiple system disorders compared to mutations in the C-terminal region [3,10,11]. R133L is a relatively common mutation in the *LMNA* rod domain; however, the mechanism by which *LMNA* R133L triggers lipodystrophy remains largely unknown.

Here, we report a new condition in a 26-year-old female, characterized by generalized lipoatrophy commencing at the onset of puberty; severe insulin resistance and diabetes; hypertriglyceridemia; a fatty liver; featuring specific, focal segmental glomerulosclerosis (FSGS); and hypertension. She harboured a novel heterozygous substitution R133L in the α-helical rod domain of *lamin A/C*. Her 6-yr-old son carried the same mutation but has not (yet) developed a lipodystrophic phenotype. We investigated the effects of *LMNA* R133L on the nuclear structures of adipocytes (3T3-L1 cells), especially in terms of adipocyte differentiation, global genomic expression, and the insulin-signalling pathway; we explored how *LMNA* R133L might affect adipose metabolism.

## Patient and methods

### Patient, her pedigree, and informed consent

The research protocol was approved by ethics committee of Sir Run Run Shaw Hospital affiliated with Medical School of Zhejiang University. The patient, both parents, her brother, her sister, and her son were referred to us and gave written informed consent for publication of their data.

### Phenotype characterization

Routine serum measurements and 75-g oral glucose tolerance tests (OGTTs) were performed after 12 h, overnight fasts. Glucose and insulin levels 0 and 2 h later were measured using enzymatic and chemiluminescent methods, respectively. Serum adiponectin and leptin levels were determined by ELISA (Millipore; Billerica, MO, USA). Fat deposition in the abdomen and thighs of the patient was assessed by computed tomography (CT).

### Genetic analysis

Genomic DNA was isolated from peripheral blood lymphocytes using a QIAamp DNA Blood Mini Kit (Qiagen; Chatsworth, CA, USA). Direct sequencing of patient genes associated with lipodystrophy and/or micrognathia, including *LMNA, ZMPSTE24, AGPAT2, BSCL2, CAV1, PTRF*, PPARγ2, and *WRN*, was performed by the BGI-Huada Genomics Institute. The R133L mutation was found in *LMNA* exon 2 of the proband; the genomic DNA of the pedigree was thus subjected to verification via Sanger sequencing.

### Immunofluorescence studies

3T3-L1 pre-adipocyte was purchased from the American Type Culture Collection (Manassas, VA, USA) and grown on coverslips (Thermo-Scientific^TM^; Waltham, MA, USA). Cells were fixed in 4% (v/v) paraformaldehyde (PAF) for 20 min, permeabilized in phosphate-buffered saline (PBS) with 0.2% (v/v) Triton X-100 for 10 min, and blocked with 3% (w/v) BSA in PBS for 30 min. Sections of abdominal skin tissue of the female patient were prepared from representative blocks of paraffin-embedded tissues, dewaxed, rehydrated, blocked with 3% (v/v) H_2_O_2_ in PBS for 10 min at room temperature, and incubated in 10% (v/v) goat serum for 30 min to block non-specific binding. Tissue sections and cells on coverslips were then incubated with a mouse monoclonal antibody against anti-lamin A/C 346 (sc-7293, Santa Cruz Biotechnology Inc.; Santa Cruz, CA, USA) or a rabbit monoclonal anti-lamin B1 antibody (Abcam; Cambridge, UK) at a dilution of 1:200 or 1:400 in 10% (v/v) goat serum overnight at 4°C. After several washes with PBS, sections were incubated with a goat anti-mouse secondary antibody (Alexa Fluor®594, Invitrogen; Grand Island, NY, USA) or a goat anti-rabbit secondary antibody (Alexa Fluor®488, Invitrogen) at a 1:1,500 dilution for 1 h at room temperature; counterstained with (496 diamidi-no-2-phenylindole [DAPI]; a nuclear counterstain); cover-slipped; and examined under a fluorescence microscope (Zeiss Germany).

### Cell culture and pre-adipocyte differentiation

3T3-L1 cells were cultured in Dulbecco’s modified Eagle’s medium (DMEM) containing 10% (v/v) fetal bovine serum (FBS) (Bio-Rad; Hercules, CA, USA) and 100 IU/mL penicillin/streptomycin at 37°C under 5% (v/v) CO_2_ at 95% relative humidity. Two-day post-confluent 3T3-L1 cells (Day 0 cells) were induced to differentiate into adipocytes by addition of a differentiation mixture (DMEM containing 10% [v/v] FBS, 10 mg/mL insulin, 0.5 mM 3-isobutyl-1-methylxanthine (IBMX), and 1 mM dexamethasone). Two days later, the culture medium was changed to DMEM supplemented with 10% (v/v) FBS and 10 mg/mL insulin for 2 days. The medium was then replaced every other day with DMEM containing 10% (v/v) FBS, and incubation continued for various times.

### Transfection of 3T3-L1 preadipocytes

cDNA encoding human lamin A/C was amplified by PCR using DNA sequence information from GenBank (accession no. NM_170707; www.ncbi.nlm.nih.gov). The R133L point mutation was introduced into full-length human lamin A/C cDNA using a QuikChange site-directed mutagenesis kit (Stratagene; La Jolla, CA, USA) and confirmed by DNA sequencing. The primers used for cDNA amplification and mutagenesis were as follows: *LMNA* WT (forward): 5ʹ-TCAGG CTCGG CTGAA GGAC-3ʹ; reverse: 5ʹ-CCAGC TTGGC AGAAT AAGTC TTC-3ʹ; and *LMNA* R133L (forward): 5ʹ-TCAGG CTCTG CTGAA GGAC-3ʹ; reverse: 5ʹ-CCAGC TTGGC AGAAT AAGTC TTC-3ʹ. Plasmids expressing FLAG-tagged *LMNA* (wild-type and R133L) were generated by inserting lamin A/C cDNA between the *Xba*I and *Eco*RI restriction sites of the pCh-EFα1-Flag-N-IRES-EGFP-2 (pCH-Flag-N2) lentivirus vector containing the human EF1α promoter. Recombinant lentiviruses were produced by co-transfection of a pCH-Flag-N2 plasmid containing cDNA encoding *LMNA* wild-type or R133L, the psPAX2 packaging plasmid, and the pMD2G enveloping plasmid (ratio 4:3:1 by weight) into 293T cells (at 80–90% confluence) using the ExFect reagent (ExcellBiology; Shanghai, China). Viral transduction efficiencies were determined by evaluating the expression levels of green fluorescent protein under a fluorescence microscope (Zeiss). At 48 h after transfection, media containing recombinant lentiviruses were passed through 0.45-μm-pore- diameter filters and concentrated for use in transduction experiments. In transient expression experiments, 3T3-L1 pre-adipocytes at 60–80% confluence were transduced with lentiviruses by addition of appropriate volumes of virus-containing medium (1:200) containing polybrene (6 μg/mL), followed by incubation for 12 h and three washes with fresh medium. Cells were harvested after 48 h.

3T3-L1 cells transfected with control lentivirus vector, *LMNA* wild-type, or R133L lentiviruses were selected on growth medium containing 4.0 mg/mL puromycin (Sigma-Aldrich; Saint Louis, MO, USA) for 48 h to eliminate uninfected cells. Stably transfected 3T3-L1 cells were then induced to differentiate. Efficient expression of *LMNA* wild-type or R133L in 3T3-L1 cells was verified by qRT-PCR and Western blotting.

### Onearray analysis

We used a GeneChip Mouse OneArray Plus 2.0 microarray (MOA2.1, Shanghai Genechem Co. Ltd.; Shanghai, China) to study global gene expression. 3T3-L1 cells stably expressing *LMNA* wild-type or R133L were induced to differentiate over 4 days and total RNAs isolated with TRIzol (Invitrogen) followed by purification on a Qiagen column (Qiagen; Valencia, CA, USA). RNA integrity was evaluated using the RNA 6000 nanoassay (Agilent Technologies, Palo Alto, CA, USA); RNA purity and concentration were determined using a NanoDrop ND-1000 spectrometer (NanoDrop; Wilmington, DE, USA). Subsequent data analyses were performed using the Rosetta Resolver System (Rosetta Biosoftware; Seattle, WA, USA) and David 6.8 software (http://david.abcc.ncifcrf.gov/). Data were normalized using a robust, multi-array averaging algorithm that yielded fold changes in the expression levels (log_2_|Foldchange| ≥1 at P < 0.05) of selected genes.

### Quantitative real-time RT-PCR

Total RNA was isolated using TRIzol (Invitrogen) and reverse-transcribed employing a PrimeScript^TM^ RT kit (TaKaRa; Tokyo, Japan) according to the manufacturer’s protocol. Real-time PCR was performed using an Applied Biosystems 7500 Real-Time PCR system (ABI Applied Biosystems; Foster city, CA, USA) and a GoTaq® Green Master Mix kit (Promega; Madison, WI, USA). The relative expression levels of genes of interest were normalized to those of β-actin (internal control) to allow quantification of individual mRNA species; we used the 2^−∆∆Ct^ formula. The primer sets were: *β-actin* forward: 5ʹ-TGCTG TCCCT GTATG CCTCT G-3ʹ, *β-actin* reverse: 5ʹ-TTGAT GTCAC GCACG ATTTC C-3ʹ; *PPARγ* forward: 5ʹ-TGTCG GTTTC AGAAG TGCCT TG-3ʹ; *PPARγ* reverse: 5ʹ-TTCAG CTGGT CGATA TCACT GGAG-3ʹ; *SREBP-1c* forward: 5ʹ-GGAGC CATGA TTGCA CATT-3ʹ; *SREBP-1c* reverse: 5ʹ-CCTGT CTCAC CCCCA GCATA-3ʹ; *ACC1* forward: 5ʹ- GAGCA GCCCA TTCTC ATCTA TATC −3ʹ; *ACC1* reverse: 5ʹ- CTGTG TGTGC TCTGG TTCAG CTC −3ʹ; *aP2* forward: 5ʹ- TTTGT ATCAC CCTAA CCTG −3ʹ; *aP2* reverse: 5ʹ- TGCCC TTTCG TA AAC TCT −3ʹ; *SCD1* forward: 5ʹ-GGCTA GCTAT CTCTG CGCTC-3ʹ; *SCD1* reverse: 5ʹ-GAACT GCGCT TGGAA ACCTG-3ʹ; C/EBP*α* forward: 5ʹ-CAAGA ACAGC AACGA GTACC G-3ʹ; C/EBP*α* reverse: 5ʹ-GTCAC TCGTC AACTC CAGCA C-3ʹ; *Nipbl* forward: 5ʹ- TCCCC AGTAT GACCC TGTTT −3ʹ; *Nipbl* reverse: 5ʹ-AGAAC ATTTA GCCCG TTTGG-3ʹ; *Adamts-7* forward: 5ʹ-GGAGT GAGGA CCCAG ATAAG TA-3ʹ; *Adamts-7* reverse: 5ʹ-CGTGC ATAGG TGAAG GTAGT G-3ʹ; *ECM1* forward: 5ʹ-ACCAC ATGGC TGAGT TCG-3ʹ; *ECM1* reverse: 5ʹ-AAGGC TGCTC TGGAT ACG-3ʹ; *Col4α1* forward: 5ʹ-CAAAA GGTGA CAAGG GAGAG CAAG-3ʹ; *Col4α1* reverse: 5ʹ-GCTCC CCCTT TCTCC TTTTT CA-3ʹ; *Col6α1* forward: 5ʹ-TGCAG GCATT GAGAT CTTTG −3ʹ; *Col6α1* reverse: 5ʹ-CAGGG CCTGG TAGTT TGGTA-3ʹ; *Col1α1* forward: 5ʹ-TAGGC CATTG TGTAT GCAGC-3ʹ; *Col1α1* reverse: 5ʹ-ACATG TTCAG CTTTG TGGAC C-3ʹ; *Postn* forward: 5ʹ-AACCA AGGAC CTGAA ACACG-3ʹ; *Postn* reverse: 5ʹ-GTGTC AGGAC ACGGT CAATG-3ʹ; *MMP2* forward: 5ʹ-CAACG GTCGG GAATA CAGCA G-3ʹ; *MMP2* reverse: 5ʹ-CCAGA AAGTG AAGGG GAAGA-3ʹ; *Rora* forward: 5ʹ-CAATG CCACC TACTC CTGTC C-3ʹ; *Rora* reverse: 5ʹ-GCCAG GCATT TCTGC AGC-3ʹ; and *Angpt1* forward: 5ʹ-AGGCT TGGTT TCTCG TCAGA-3ʹ; *Angpt1* reverse: 5ʹ-TCTGC ACAGT CTCGA AATGG-3ʹ.

### Western blotting

Briefly, 3T3-L1 cells stably expressing *LMNA* wild-type or R133L were induced to differentiate over 4 days. Cells were washed with PBS, followed by incubation with 100 nM insulin for 15 min to stimulate insulin signalling, and then cells were harvested for total protein and membrane protein extraction using Mem-PER Plus Kit (Thermo Scientific™; Waltham, MA, USA). Equal amounts of protein were boiled and separated via SDS-polyacrylamide gel electrophoresis; transferred to Immun-Blot PVDF membranes (Millipore, Billerica, MO, USA); blocked with 5% (w/v) non-fat milk for 1 h at room temperature; incubated with primary antibodies (diluted 1:1,000) including anti-rabbit antibodies against PI3K, p-Akt^473^, p-Akt^308^, Akt, PPARγ, β-actin, Flag, histones (Cell Signaling Technology Inc., Danvers, MA, USA) and the Glut-4 plasma membrane protein (Abcam); and a 1:1,000 dilution of an anti-mouse antibody against lamin A/C (Santa Cruz Biotechnology) at 4°C overnight. After subsequent incubation with horseradish-peroxidase-conjugated goat anti-rabbit/mouse secondary antibodies at room temperature for 1 h, immunoreactive proteins were detected using an electrochemiluminescence kit (Millipore, Billerica, MO, USA).

### Oil red O-staining

Oil Red working solutions (Jiancheng, Nanjing, China) were filtered through 0.2-mm-pore-diameter filters. Cells of 10 days differentiation were fixed in 4% (v/v) formalin for 15 min at room temperature, washed with 60% isopropanol, allowed to dry, incubated with Oil Red O working solution for 1 h, and washed several times with PBS.

### Statistical analysis

Data are expressed as means ± SDs. Differences between individual group means were compared using the independent t-test or one-way ANOVA followed by the least-significant difference, multiple range test. A P-value < 0.05 was considered to reflect statistical significance. All experiments were performed in triplicate.

## Results

### Case report

**Figure 1.**
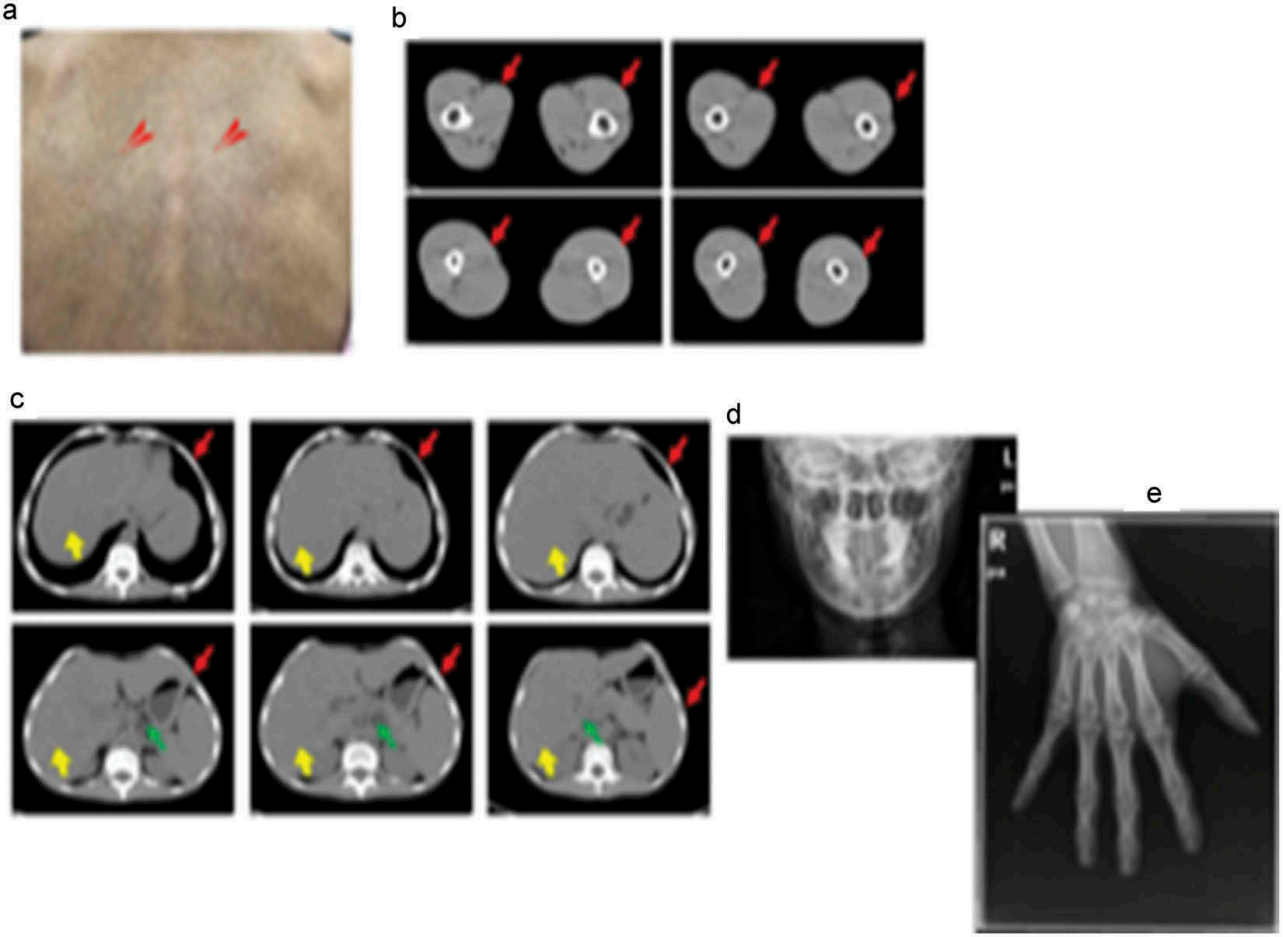
Clinical characteristics of laminopathy in this patient. (a). Mottled hyper-and hypo-pigmentation of the back skin (red arrows). (b). Loss of thigh subcutaneous fat as revealed by computed tomography (red arrows). (c). Liver hypertrophy (yellow arrows) and loss of abdominal subcutaneous (red arrows) and visceral fat (green arrows) as revealed by computed tomography. (d). Micrognathia. (e). Absence of finger acro-osteolysis.

**Figure 2.**
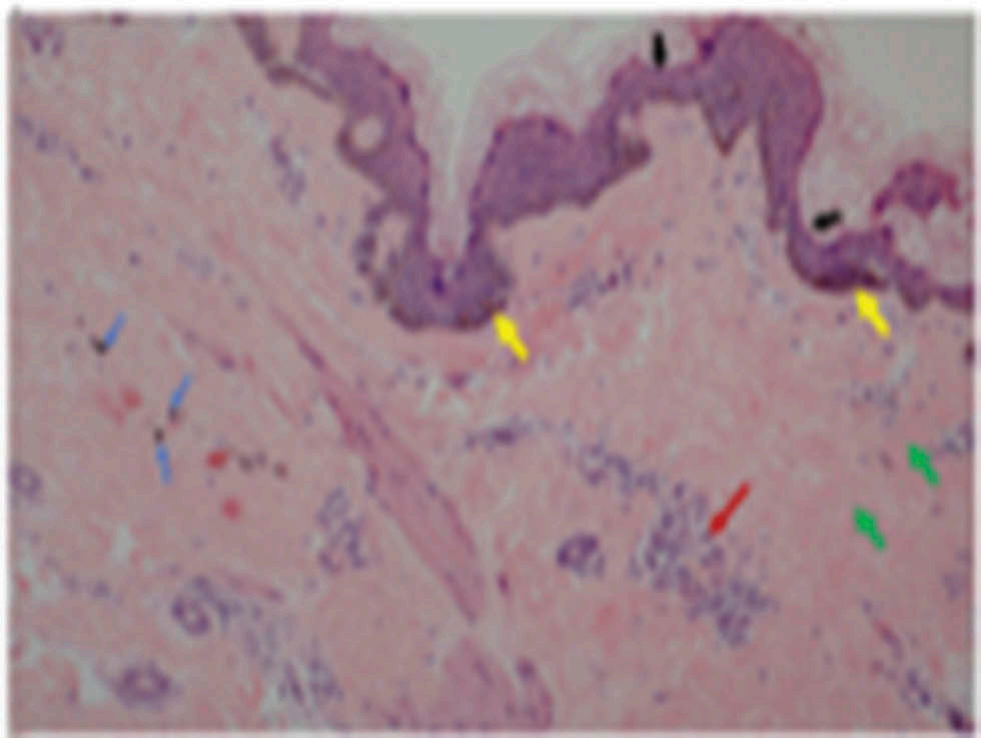
Abdominal skin biopsy findings of the female patient (100X). Slight epidermal atrophy (black arrows) and increased pigmentation in the basal layer of skin (yellow arrows) and pigment incontinence (blue arrows) and dermal collagen compact (green arrows) and infiltration of perivascular inflammatory cells (red arrows).

A 26-year-old Han Chinese female was of low birth weight (1.8 kg) as one of twins; the other twin (a boy of birth weight 2.5 kg) died at the age of 3 years. Around the time of puberty, she noticed progressive fat loss from the face, extremities, and abdomen. Although she reported normal menarche and a regular menstrual cycle, her breast development was poor. She had a small chin associated with dental overcrowding. She was diagnosed with diabetes at the age of 22 years, hypertension at the age of 23 years (during pregnancy) and underwent a forced abortion at 7 months of pregnancy. She was further diagnosed with overt proteinuria at the age of 25 years. She had been receiving insulin therapy for 3 years at doses titered up to 100 U daily combined with 1,500 mg metformin daily, but her glucose control was poor; the HbA1c level was about 9.0%. She was taking 150 mg Irbesartan daily to treat hypertension and proteinuria. On physical examination, she was 149 cm tall and weighed 33.5 kg (body mass index, 15.09 kg/m^2^). She had a small face with sunken cheeks, a small chin, a beaked nose, a high forehead, and marked thinning of the eyebrows. She had thin, sparse, curly, progressively greying hair. She had minimal breasts (Tanner stage III). She lacked eyebrow, axillary and pubic hair. A generalized loss of subcutaneous fat was evident, involving the face, trunk, and extremities; the subcutaneous veins were prominent on the back of the feet and hands, and the upper limbs and back. Her body fat percentage was 10.3% (normal 25–28%) as measured using a portable, bioelectrical impedance analyser (Roche, Basel, Switzerland). Mottled hyper- and hypo-pigmentation was evident on the neck, back, extremities, and abdomen, but no acanthosis nigricans were recorded (Figure 1(a)). Laboratory measurements revealed that her glucose levels were uncontrolled. The oral glucose tolerance test revealed marked basal and post-loading hyperinsulinemia. She exhibited hypertriglyceridemia, hepatomegaly, and elevated alanine and aspartate aminotransferase levels (Table 1) indicative of a fatty liver, as confirmed by ultrasound and abdominal CT. She evidenced overt proteinuria but normal serum creatinine levels and a normal fundus on ophthalmoscopic examination. CT of the thighs and abdomen revealed significant loss of subcutaneous and visceral fat for example, the perirenal region was almost free of fat, Figure. 1(b-c), and fatty hepatomegaly. The serum adiponectin and leptin concentrations were low, at 0.654 μg/mL and 1.356 ng/mL (range of serum leptin levels in normal lean woman: 3.7–11.1 ng/mL), thus significantly lower than those of her female pedigree (mean adiponectin level 4.52 μg/mL and mean leptin level 21.41 ng/mL, respectively) (Table 1). Ultrasound revealed atherosclerotic plaque in the left internal carotid artery and the tibial arteries. Echocardiography revealed left ventricular hypertrophy with decreased ventricular diastolic capacity. Micrognathia (but no acro-osteolysis) was evident in the terminal phalanges and clavicular region, as revealed by X-rays Figure. 1(d-e). An abdominal skin biopsy was performed; histological examination revealed slight epidermal atrophy, increased pigmentation in the basal layer of skin, pigment incontinence, dermal collagen compact and infiltration of perivascular inflammatory cells Figure 2. Renal biopsy and histological examination revealed focal segmental glomerulosclerosis (FSGS) (data unpublished).

**Table 1.** Characteristics of the pedigree.

Characteristics	Proband	Father	Mother	Sister	Brother	Son
Height, cm	149	163	162	153	168	113(-2SD~-1SD)
Weight, kg	33.5	50	60	55	67	16.5(-1SD)
BMI, kg/m^2^	15.09	18.81	22.86	23.49	23.73	
FBS, mmol/L	9.00	4.20	3.98	4.82	3.64	4.37
PBS, mmol/L	16.13	4.28	3.39	8.68	4.85	5.62
FIns, μIU/mL	20.20	2.20	4.22	7.61	3.63	2.19
2h-Ins, μIU/mL	139.63	5.98	13.70	75.37	29.31	6.85
Adiponectin, μg/mL	1.356	13.00	6.591	2.429	5.536	7.874
Leptin, ng/ml	0.654	1.009	17.15	25.41	4.309	2.711
TG, mmol/L	5.21	1.14	0.91	1.05	0.68	0.41
TC, mmol/L	3.55	4.75	4.96	5.80	5.59	3.45
LDL-c, mmol/L	1.66	3.19	2.91	4.00	4.13	1.71
HDL-c, mmol/L	0.67	0.98	1.09	1.05	0.89	1.33
VLDL-c, mmol/L	1.16	0.55	0.80	0.65	0.52	0.35
GPT, U/L	86	13	17	13	22	8
GOT, U/L	59	22	23	19	23	29
γGT, U/L	97	19	18	15	20	6
ALP, U/L	74	85	67	62	84	238
TBil, μmol/L	8.0	11.0	9.1	11.3	8.1	2.9
DBil, μmol/L	2.7	2.7	2.9	2.9	2.6	0.2

Abbreviations: BMI, body mass index; FBS, fasting blood sugar; PBS, 2 h postprandial blood sugar; Fins, fasting insulin levels; 2h-Ins, 2 h postprandial insulin levels; TG, triglyceride; TC, total cholesterol; LDL-c, low density lipoprotein cholesterol; HDL-c, high density lipoprotein cholesterol; VLDL-c, very low density lipoprotein cholesterol; GPT, glutamic-pyruvic transaminase; GOT, glutamic-oxal(o) acetic transaminase;γGT,γ-glutamyl transaminase; ALP, alkaline phosphatase; TBil, total bilirubin; DBil, direct bilirubin.

### Characteristics and genetic analysis of the pedigree

**Figure 3.**
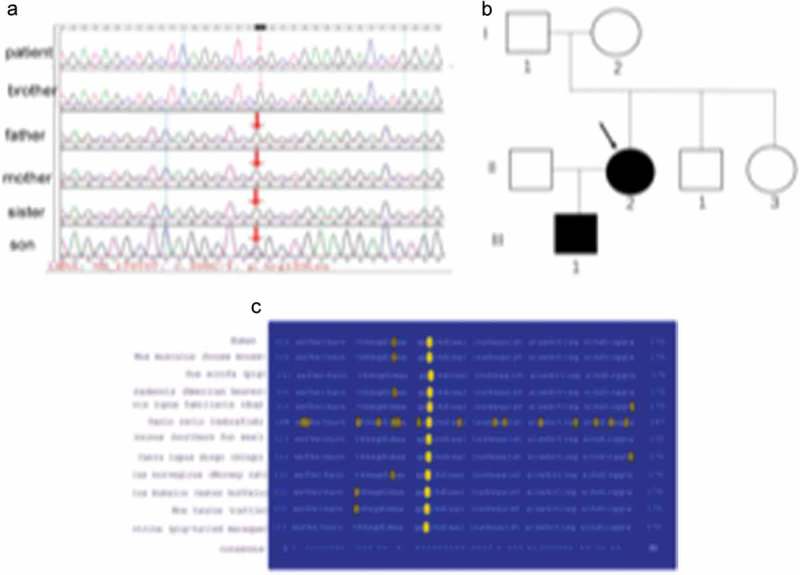
LMNA gene analysis in the pedigree. (a). Genetic analysis of the *LMNA* gene of the patient and her pedigree. (b). The search for the *LMNA* R133L mutation in the patient’s relatives (arrow: proband). (c). Sequence conservation of R133 in vertebrate *LMNA* data in the Swiss-Prot database (with accession numbers) and alignments around the conserved R133 residue (red).

The pedigree of the patient consisted of both parents, one sister, one brother, and her 6-year-old son. All five individuals were clinically examined, and none evidenced lipodystrophy, hepatomegaly, or skin lesions. Laboratory measurements, including the 75-g oral glucose tolerance test and the insulin response (the fasting and 2-h glucose and insulin levels), and the levels of triglycerides and hepatic enzymes, were normal (Table 1). We directly sequenced all exons of genes associated with lipodystrophy including *LMNA, ZMPSTE24, AGPAT2, BSCL2, CAV1, PTRF*, and PPARγ2. We identified a heterozygous CGG to CTG transition at *LMNA* codon 133 c.398G>T, exon 2, Figure 3(a) triggering an arginine-to-leucine substitution (R133L). Of her family members, the mutation was present in only her 6-year-old son, who lacked lipodystrophy at the time of evaluation Figure 3(b). The absence of the mutation in her parents, brother, and sister indicate that the mutation in this patient was sporadic and de novo. This residue is highly conserved in vertebrate *lamins A/C* Figure 3(c).

### Cell studies

#### Nuclear morphologies of skin tissues of the patient, and of 3T3-L1 pre-adipocytes overexpressing LMNA R133L

**Figure 4.**
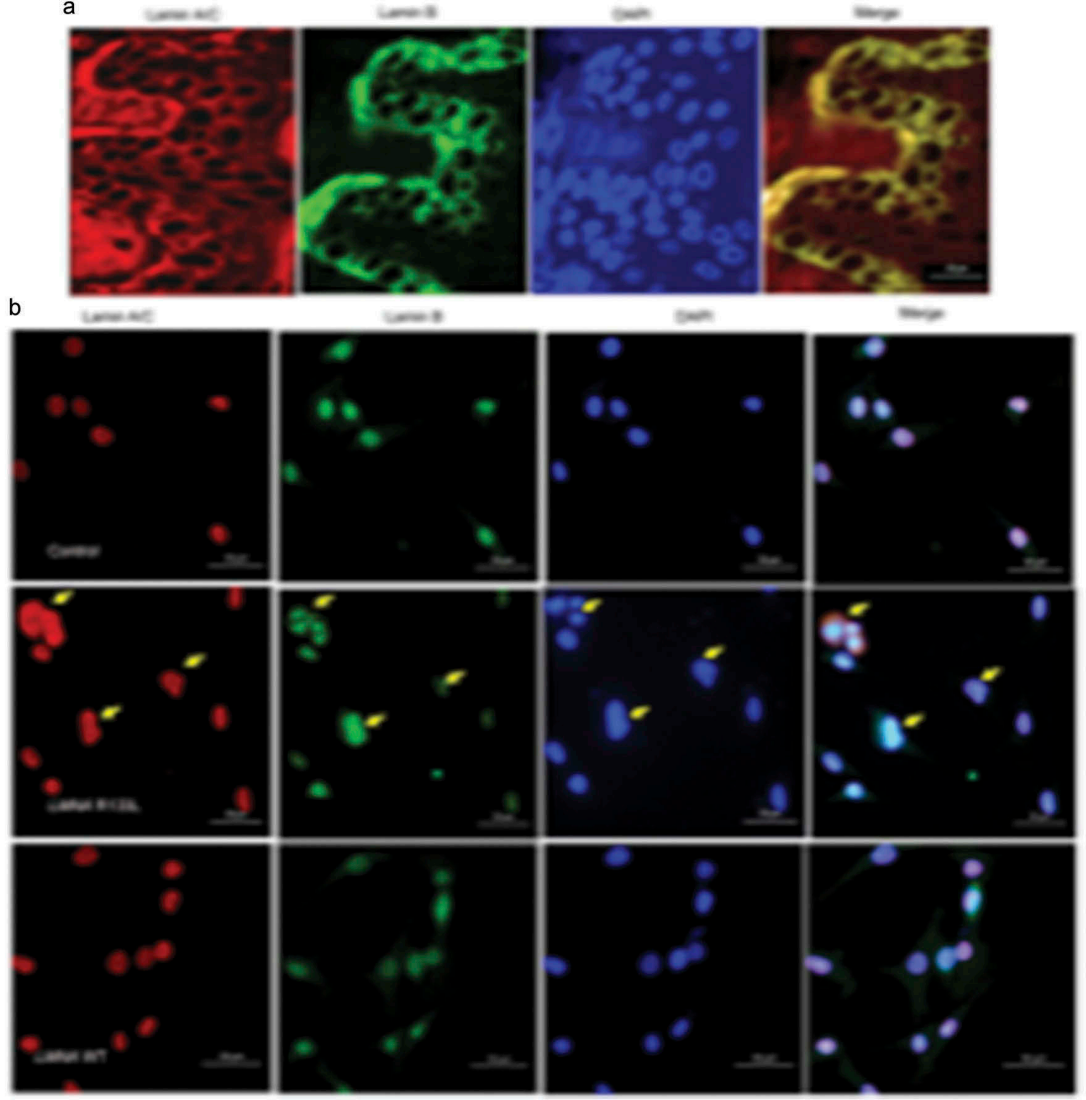
Nuclear morphology and immunohistological staining of lamins in skin tissue and pre-adipocytes. (a). Immunohistological staining of lamins in abdominal skin biopsies from the female patient. (b). Abnormal features of the nuclear envelope in 3T3-L1 pre-a dipocytes transfected with wild-type *LMNA* or the R133L mutant *LMNA* (400X, arrows point to different nuclei abnormalities).

As the nuclear lamina is critical in terms of nuclear structure and organization, we studied the nuclei of abdominal skin tissues of the female patient; we used double immunofluorescence staining to visualize *lamin A/C* and its principal partner, *lamin B*, in the nuclear envelope. No abnormally shaped nuclei were apparent in skin biopsies from the patient. Both *lamin A/C* and *lamin B* were homogeneously distributed over the entire nuclear peripheries, and co-localized, as evident in merged images Figure 4(a). To clarify the pathogenic role played by the *LMNA* R133L mutation in lipodystrophy, we analysed the nuclear shapes of 3T3-L1 pre-adipocytes overexpressing *LMNA* wild-type or *LMNA* R133L. Nuclear morphologies varied from normal to very abnormal. In control lentivirus transfected cells and cells overexpressing *LMNA* wild-type, the nuclei of 3T3-L1 cells were round and stained homogeneously for both *lamin A/C* and *lamin B*, which were distributed around the entire nuclear periphery and co-localized. In contrast, several abnormalities in terms of nuclear shape and lamin protein distributions were observed in cells overexpressing *LMNA* R133L. In 40–50% of pre-adipocytes overexpressing the mutant protein, the nuclei showed disturbed shapes, varied in size, and exhibited nuclear membrane invagination or blebbing. Immunofluorescence staining revealed that the A-type lamin meshwork was irregularly distributed around some nuclear envelopes. *Lamin B* staining was also be in-homogeneous in *LMNA* R133L cells compared to control or *LMNA* wild-type cells Figure 4(b).

#### Effects of LMNA R133L overexpression on differentiation of 3T3-L1 pre-adipocytes and the insulin-signalling pathway

**Figure 5.**
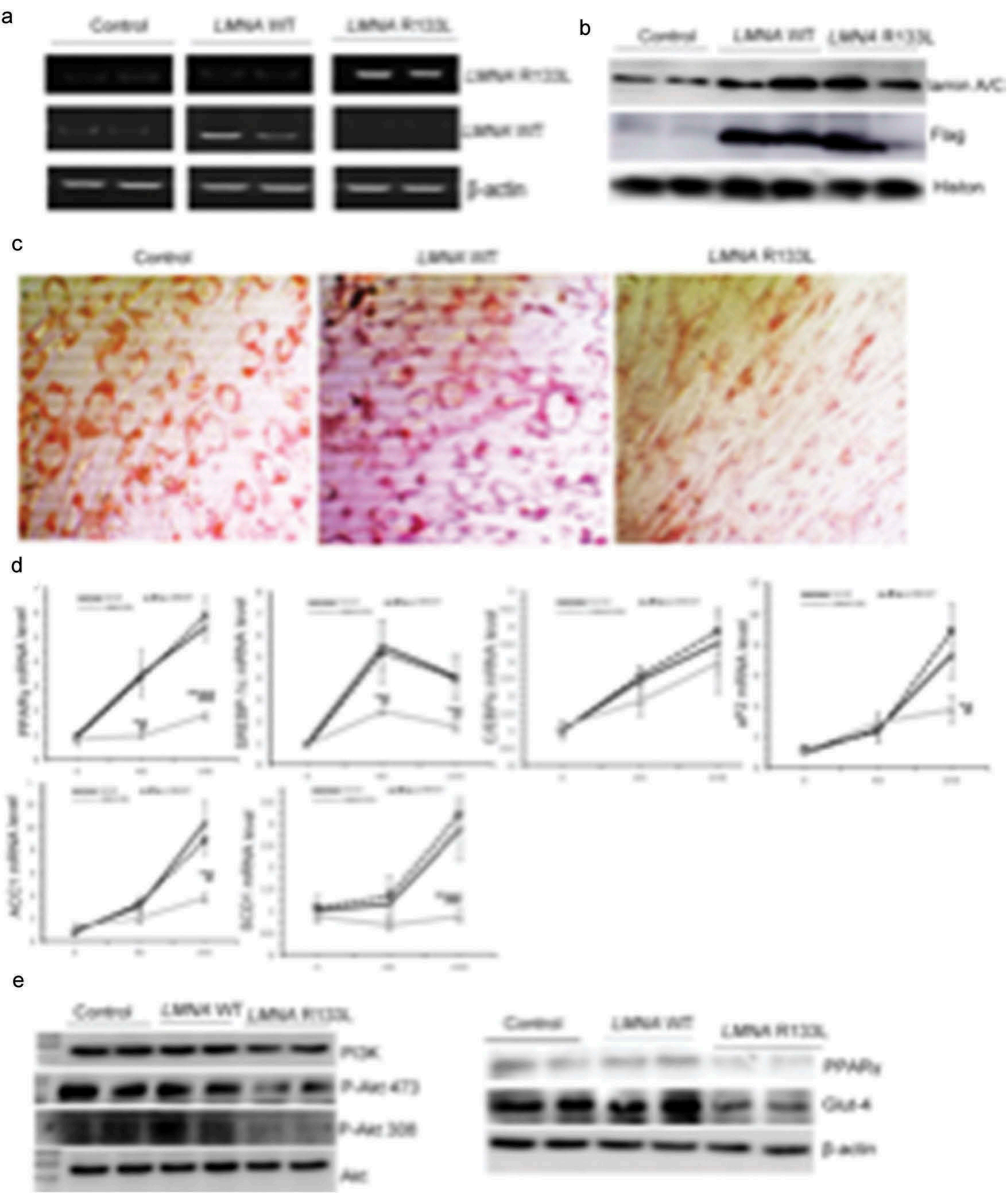
Stable expression of human *LMNA* R133L impairs adipocyte differentiation and insulin pathway signalling. (a). Expression of human *LMNA* wild-type or *LMNA* R133L as detected by RT-PCR in three clonal lines of 3T3-L1 cells transfected with control lentivirus, or lentivirus encoding Flag-tagged human *LMNA* wild-type or *LMNA* R133L. (b). Western blotting of *lamin A/C* and Flag-tagged proteins in three clonal lines of 3T3-L1 cells. (c). Red-Oil staining of three clonal lines of 3T3-L1 cells after 10 days of differentiation (200 X). (d). Expressions of adipogenic or lipogenic genes in each of three clonal lines of 3T3-L1 cells transfected with control lentivirus, or lentivirus encoding Flag-tagged human *LMNA* wild-type or *LMNA* R133L induced over 4 days and 10 days differentiation as revealed by qPCR using gene-specific primers (n = 3). Data are presented as means ± SDs. *P < 0.05 vs. Control; **P < 0.01 vs. Control. ^#^P < 0.05 vs. *LMNA* wild-type; ^##^P < 0.01 vs. *LMNA* wild-type. (e). PI3K/Akt signalling pathway activity upon insulin treatment of three clonal lines of 3T3-L1 cells after 4 days of differentiation.

To explore the effects of the *LMNA* R133L mutation on adipocyte differentiation, we established three clonal lines (each) of 3T3-L1 cells transfected with control lentivirus, and lentivirus encoding Flag-tagged human *LMNA* wild-type or *LMNA* R133L. Expression of the human *LMNA* wild-type or *LMNA* R133L mutation in 3T3-L1 cells was confirmed by RT-PCR using primers targeting the wild-type or the R133L mutation site, respectively (Figure 5(a)). *LMNA* mRNA bearing the R133L mutation was detected only in cells transfected with the *LMNA* R133L lentivirus, thus not in cells transfected with *LMNA* wild-type or control lentivirus. Western blotting revealed that *lamin A/C* protein was overexpressed by 3T3-L1 cells transfected with lentivirus encoding either *LMNA* wild-type or *LMNA* R133L, but not control cells Figure 5(b). The Flag tag was detected only in cells transfected with *LMNA* wild-type or *LMNA* R133L, indicating successful expression of the Flag-tagged human *LMNA* wild-type or *LMNA* R133L protein in 3T3-L1 cells Figure 5(b).

Control 3T3-L1 cells or cells stably expressing *LMNA* wild-type exhibited many intracellular lipid droplets (revealed by Oil-Red staining) after 10 d of differentiation. In contrast, 3T3-L1 cells stably expressing *LMNA* R133L were unable to accumulate lipids Figure 5(c). Accordingly, transcriptional factors participated in adipogenesis including *PPAR*γ and SREBP-1c were significantly inhibited in 4-day- and 10-day-differentiated cells, whereas, pro-lipogenic marker genes of aP2, SCD1 and ACC1 were markedly inhibited in 10 days-differentiated adipocytes stably expressing *LMNA* R133L Figure 5(d), indicating that differentiation was impaired by the *LMNA* R133L mutation. We further examined the effect of this mutation on insulin-signalling by 4-day-differentiated 3T3-L1 cells. Insulin-mediated activation of both the PI3K/Akt pathway and (downstream) associated Glut-4 membrane translocation was significantly suppressed in *LMNA* R133L cells. The expression of PPARγ, a key regulator of differentiation, was also suppressed by the *LMNA* R133L mutation Figure 5(e).

#### Gene transcriptome of 3T3-L1 pre-adipocytes overexpressing LMNA R133L after 4 days of differentiation

**Figure 6.**
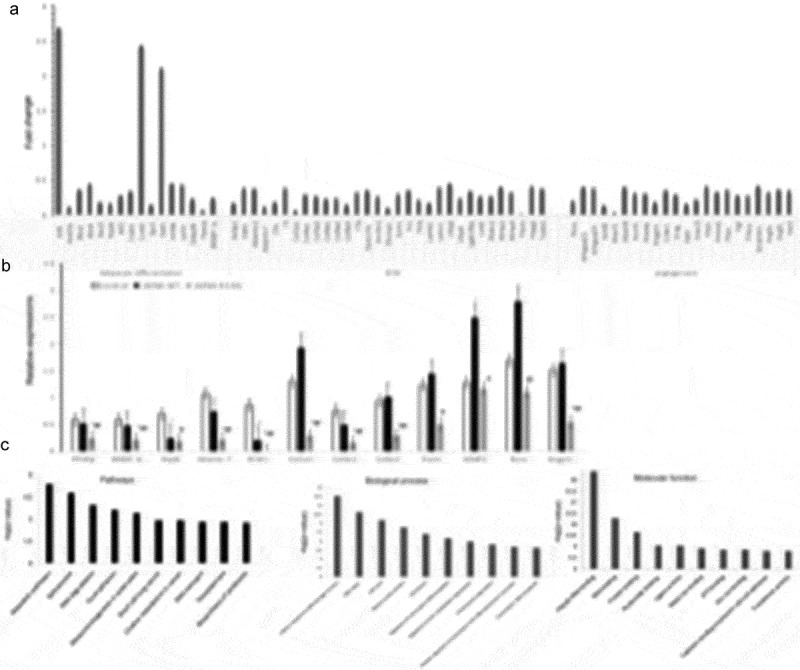
The transcriptome and gene expressions in adipocytes overexpressing *LMNA* R133L. (a). The transcriptome associated with adipocyte differentiation of 3T3-L1 cells. (b). Validation of the Affymetrix gene expression data in each of three clonal lines of 3T3-L1 cells transfected with control lentivirus, or lentivirus encoding Flag-tagged human *LMNA* wild-type or *LMNA* R133L, as revealed by qPCR using gene-specific primers (n = 3). Data are presented as means ± SDs. *P < 0.05 vs. Control; **P < 0.01 vs. Control. ^#^P < 0.05 vs. *LMNA* wild-type; ^##^P < 0.01 vs. *LMNA* wild-type. (c). Ingenuity pathway, molecular functional, and biological process analyses of 3T3-L1 cells stably expressing *LMNA* wild-type or *LMNA* R133L.

We speculated that the laminar abnormalities caused by the *LMNA* R133L mutation might exert downstream effects on genomic expression, which might in turn be associated with multiple system disorders triggering laminopathy. We examined the transcriptomes of 4-day-differentiated 3T3-L1 cells stably expressing *LMNA* wild-type or R133L. We found that the expression levels of key genes implicated in adipocyte differentiation (including *PPARγ, SREBP-1c*, and *BSCL2*) were decreased. Furthermore, we found that the expression of genes encoding extracellular matrix components was inhibited, including collagen types 1, 4, 5 and 6, laminin, fibronectin, *MMP2* and *MMP9*, and genes involved in angiogenesis, including *PTEN* and *VEGF-A* and *–C* Figure 6(a). We used qRT-PCR to confirm that the *PPARγ, SREBP-1c*, and *Nipbl* genes; the extracellular matrix genes encoding *Col1α1, Col4α1, Col6α1, MMP2*, and *Adamts-7*; and the angiogenesis genes encoding *Angpt1* and *Rora*, were significantly downregulated in *LMNA* R133L cells compared to control and *LMNA* wild-type cells Figure 6(b).

We employed the David ver. 6.8 database to identify the metabolic pathways and molecular functions compromised by changes in the levels of mRNAs encoding proteins affected by the *LMNA* R133L mutation. The most prominent difference (indicated by KEGG pathway analysis) between cells bearing *LMNA* wild-type and the R133L mutant was in genes implicated in metabolic pathways. The most prominent differences in terms of the gene ontologies of molecular function and biological processes were in genes implicated in RNA metabolism (poly-A RNA) binding, the cellular response to DNA damage, and DNA repair Figure 6(c).

## Discussion

We describe a new laminopathy associated with mandibuloacral dysplasia, generalized lipoatrophy developing at the onset of puberty, insulin-resistant diabetes, hypertriglyceridemia, liver steatosis, hypertension, and biopsy-proven, focal segmental glomerulosclerosis in a patient affected by a novel R133L heterozygous substitution in the dimerization rod domain of *lamin A/C*. The mutation was sporadic, being absent in the patient’s parents. Unfortunately, her son carries the mutation.

Co-existence of mandibuloacral dysplasia and focal segmental glomerulosclerosis has been reported in two patients with ZMPSTE24 deficiencies [12]. We report the first patient in whom the *LMNA* R133L mutation is associated with FSGS; the mutation is uniquely located in the central rod domain of the N-terminal half of the *LMNA* gene. Furthermore, our *in vitro* study revealed that mouse glomerular mesangial cells overexpressing human *LMNA* R133L also exhibited nuclear abnormalities and loss of laminar integrity (data unpublished). Our data suggest that a *LMNA* missense mutation may cause renal disease via nuclear abnormality with associated genomic instability, but further investigation is required.

A single R133L point mutation in the *LMNA* gene was first reported in 2003 in a 30-year-old male, who had a phenotype similar to that of our present patient. However, he suffered from hypertrophic cardiomyopathy with valvular involvement, not glomerulosclerosis [10]. Arginine 133 is located in a charged peptide region that is highly conserved in the *lamin A/C* proteins of vertebrates. A switch from positively charged arginine to hydrophobic leucine in the dimerization rod domain of *lamin A* and *C* would be expected to impair *lamin A/C* polymerization and subsequent filament assembly, and might be associated with a more severe phenotype. Overall, generalized rather than partial lipodystrophy, and a multisystem disorder rather than specific adipose tissue involvement, was evident in patients with mutations in the central rod domain of *LMNA*, thus not in the C-terminal *LMNA* region.

In subjects exhibiting normal adipose tissue development and function, adipocytes proliferate and differentiate into lipid-containing cells that also exert an endocrine function. The circulating leptin and adiponectin levels of our patient were significantly lower than those of other females in her family, reflecting fat loss, which may be one of the principal mechanisms by which lipodystrophy triggers insulin-resistant diabetes. Her son (with the same *LMNA* R133L mutation) evidences no sign of fat loss and presently exhibits normal levels of leptin, adiponectin, and glucose; moreover, comparisons with other family members support the correlations among lipodystrophy, reduced adipokine levels, and diabetes in our patient. During adipose tissue development, *PPARγ* serves as a master regulator of adipocyte differentiation. In addition, both extracellular matrix deposition and angiogenesis are essential to maintain cell and tissue function [13–17]. However, genes promoting adipocyte differentiation, extracellular matrix deposition, and angiogenesis were consistently repressed in 3T3-L1 cells overexpressing *LMNA* R133L when the cells were grown in differentiation medium. Thus, considering that both the patient and her son were born normal, and that lipodystrophy developed in the patient after the onset of puberty, our in *vitro* results support the idea that lipodystrophy caused by the *LMNA* R133L mutation is most likely attributable to an inadequacy in the capacity of adipocytes to self-renew, as was apparent in FPLD-transgenic mice with the *LMNA* R482Q mutation [4]. Impaired adipocyte differentiation was also evident in pluripotent stem cells (iPSCs) derived from Hutchinson-Gilford Progeria Syndrome (HGPS) cells by Xiong et al. [18] and in 3T3-L1 cells stably expressing *LMNA* R482Q or R482W [19], supporting a critical role for *LMNA* in adipocyte differentiation [20]. Moreover, the insulin-signalling pathway, PI3K-Akt activity, and induced glut-4 membrane translocation were suppressed in *LMNA* R133L overexpressing adipocytes, suggesting that, in addition to the reduced whole-body insulin sensitivity caused by low levels of adipokines, insulin resistance is a response to dysplastic adipose tissue per se. In line with this suggestion, the global gene profiling data indicated that the most prominent differences evident on KEGG pathway analyses of cells bearing *LMNA* wild-type or R133L were in genes implicated in metabolic pathways.

Non-laminopathic syndromes featuring premature ageing have been linked to defects in DNA repair, as is also the case in patients exhibiting lipodystrophy and insulin resistance. Werner syndrome features inactivating biallelic mutations of WRN, a DNA helicase [21]. A recently described multisystem disorder is attributable to heterozygous mutations in POLD1, encoding DNA polymerase-delta, which cooperates with WRN to maintain genomic stability [22]. In 3T3-L1 cells bearing *LMNA* R133L, the expression levels of genes implicated in the cellular response to DNA damage/DNA repair were greatly reduced, which may contribute to the cellular senescence noted in our present patient, and to the progression of other laminopathies [5,23]. Our observations favour the existence of a pathophysiological linkage between cellular senescence, DNA damage, and lipodystrophy.

In conclusion, we describe a new phenotype in a patient affected by a novel R133L heterozygous substitution in the *lamin A/C* gene. *LMNA* R133L was associated with nuclear abnormalities in 3T3-L1 pre-adipocyte and mouse glomerular mesangial cells, impaired adipocyte differentiation, and (downstream) effects on chromatin structure/gene expression in 3T3-L1 cells; these in turn affected both the induction and repair of DNA damage. This suggests a possible link between ageing, DNA damage, and lipodystrophy. As genotype/phenotype relationships are particularly complex in *LMNA*-associated diseases, the discovery of a new mutation in *LMNA* requires further extensive clinical and molecular investigation.
